# Mouse methylation profiles for leukocyte cell types, and estimation of leukocyte fractions in inflamed gastrointestinal DNA samples

**DOI:** 10.1371/journal.pone.0290034

**Published:** 2023-10-05

**Authors:** Kazuhiro Nishiyama, Hitomi Nishinakamura, Hideyuki Takeshima, Liu Yuyu, Chihiro Takeuchi, Naoko Hattori, Haruna Takeda, Satoshi Yamashita, Mika Wakabayashi, Kotomi Sato, Kazutaka Obama, Toshikazu Ushijima

**Affiliations:** 1 Division of Epigenomics, National Cancer Center Research Institute, Tokyo, Japan; 2 Division of Surgery, University of Kyoto, Kyoto, Japan; 3 Division of Cancer Immunology, Research Institute/Exploratory Oncology Research & Clinical Trial Center (EPOC), National Cancer Center, Tokyo, Chiba, Japan; 4 Department of Epigenomics, Institute for Advanced Life Sciences, Hoshi University, Tokyo, Japan; 5 Laboratory of Molecular Genetics, National Cancer Center Research Institute, Tokyo, Japan; 6 Department of Life Engineering, Faculty of Engineering, Maebashi Institute of Technology, Maebashi, Japan; University of Michigan Medical School, UNITED STATES

## Abstract

Precise analysis of tissue DNA and RNA samples is often hampered by contaminating non-target cells whose amounts are highly variable. DNA methylation profiles are specific to cell types, and can be utilized for assessment of the fraction of such contaminating non-target cells. Here, we aimed 1) to identify methylation profiles specific to multiple types of mouse leukocytes, and 2) to estimate the fraction of leukocytes infiltrating inflamed tissues using DNA samples. First, genome-wide DNA methylation analysis was conducted for three myeloid-lineage cells and four lymphoid-lineage cells isolated by fluorescence-activated cell sorting after magnetic-activated cell sorting from leukocytes in the spleen. Clustering analysis using CpG sites within enhancers separated the three myeloid-lineage cells and four lymphoid-lineage cells while that using promoter CpG islands (TSS200CGIs) did not. Among the 266,108 CpG sites analyzed, one CpG site was specifically hypermethylated (β value ≥ 0.7) in B cells, and four, seven, 183, and 34 CpG sites were specifically hypomethylated (β value < 0.2) in CD4^+^ T cells, CD8^+^ T cells, B cells, and NK cells, respectively. Importantly, cell type-specific hypomethylated CpG sites were located at genes involved in cell type-specific biological functions. Then, marker CpG sites to estimate the leukocyte fraction in a tissue with leukocyte infiltration were selected, and an estimation algorithm was established. The fractions of infiltrating leukocytes were estimated to be 1.6–12.4% in the stomach (n = 10) with *Helicobacter pylori*-induced inflammation and 1.5–4.3% in the colon with dextran sulfate sodium-induced colitis (n = 4), and the fractions were highly correlated with those estimated histologically using Cd45-stained tissue sections [R = 0.811 (*p* = 0.004)]. These results showed that mouse methylation profiles at CpG sites within enhancers reflected leukocyte cell lineages, and the use of marker CpG sites successfully estimated the leukocyte fraction in inflamed gastric and colon tissues.

## Introduction

Precise analysis of mutations, methylation, and gene expression of epithelial cells using tissue DNA and RNA samples is often hampered by contaminating non-target cells, such as leukocytes and fibroblasts. Especially, various types of leukocytes heavily infiltrate tissues with acute and chronic inflammation [1–5]. To assess the degree of leukocyte contamination, histological analysis using formalin-fixed paraffin embedded (FFPE) samples is often conducted, but the histological section and the sample from which DNA or RNA is extracted may not necessarily have the same degrees of infiltration. Purification of epithelial cells by flow cytometry analysis enables analysis of pure epithelial cells, and single-cell RNA-seq analysis of fresh tissue samples enables extraction of gene expression in epithelial cells. However, these techniques are time-consuming and costly, and the availability of fresh tissue samples may be limited. Therefore, assessment of the fraction of infiltrating leukocytes using tissue DNA samples is needed for many molecular analyses of tissue DNA and RNA samples.

Specific cell types are known to be reflected in DNA methylation profiles, which include specific CpG sites with specific methylation statuses among cell types [6–15]. By utilizing these cell type-specific DNA methylation profiles, it is reported that cellular composition within heterogeneous DNA samples can be calculated [16–21]. Indeed, DNA methylation profiles were successfully used to identify the tissue origin of cancers of unknown primary [22]. Genomic regions differentially methylated among cell types, namely differentially methylated regions (DMRs), have been shown to be likely to overlap with gene regulatory elements, such as enhancers [7, 23]. For instance, hypomethylated DMRs in the aorta overlapped with aorta-specific superenhancers [24]. Also, fetal brain, breast, and blood-specific hypomethylated DMRs are reported to be associated with genes related to specific functions in the corresponding tissues [25]. This raises a promising possibility that cell type-specific DNA methylation can be utilized for the measurement of the fraction of infiltrating leukocytes within a tissue DNA sample. Importantly, such attempts are being conducted in humans [26, 27], and web tools, such as EpiDISH, is available [28, 29]. However, few are ongoing in mice, a very important animal model.

In this study, we aimed 1) to identify methylation profiles specific to multiple types of mouse leukocytes, and 2) to establish an algorithm that can estimate the fraction of leukocytes infiltrating tissues using DNA samples.

## Materials and methods

### Isolation of individual types of leukocytes from mouse splenocytes

Eight-week-old C57BL/6J mice were purchased from Charles River Laboratories Japan (Yokohama, Japan). Spleens dissected from mice were dissociated by gentle mashing with a syringe plunger and filtered through a 70 μm mesh (BD biosciences, Franklin Lakes, NJ). Spleen cell suspension was centrifuged for 5 min at 400×g, and red blood cells were lysed with RBC Lysis Solution (Qiagen, Venlo, Netherlands). To isolate dendritic cells (from pooled spleens of 16 mice), monocytes (from pooled spleens of 5 mice), and neutrophils (from pooled spleens of 8 mice), CD4^+^ T cells (from the spleen of one mouse), CD8^+^ T cells (from the spleen of one mouse), B cells (from the spleen of one mouse), and NK cells (from pooled spleens of 4 mice), the spleen cell suspension was mixed with MicroBeads specific for individual types of leukocytes (Miltenyi Biotec, Bergisch Gladbach, Germany). Then, specific types of leukocytes were obtained by positive selection on a MACS column. Purity of the isolated specific leukocytes was evaluated using a BD FACSMelody cell sorter (BD Biosciences) with specific antibodies [dendritic cells (CD3^-^ CD11c^+^ I-Ab^+^), monocytes (CD3^-^ CD11b^+^), neutrophils (CD3^-^, CD11b^+^, Gr1^+^), CD4^+^ T cells (CD3^+^, CD4^+^), CD8^+^ T cells (CD3^+^, CD8a^+^), B cells (CD3^+^, B220^+^), and NK cells (CD3^-^ NK1.1^+^)] (S1 Fig). The flow cytometry data obtained were analyzed by FlowJo software (Treestar, Ashland, OR). All the animal experiments were conducted under the approval of the institutional Committee for Ethics in Animal Experimentation at the National Cancer Center and the Hoshi University. The mice were sacrificed by cervical dislocation.

### Isolation of inflamed mouse gastric and colon epithelium

To obtain inflamed mouse gastric epithelium, male C57BL/6 mice were infected with *Helicobacter pylori* (*H*. *pylori*) (PMSS1 strain) [2] at 3 or 20 weeks of age, and the stomach was resected after 12-week infection. From the resected stomach, gastric epithelium was scraped out from the antral and corpus regions. To obtain inflamed mouse colonic epithelium, female C57BL/6 mice were administrated with 2.5% (w/v) dextran sulfate sodium (DSS) in drinking water for 5 days. After an interval of 2 weeks, mice were administrated with 2.5% (w/v) DSS again for 5 days. Colonic epithelium was isolated from the entire colon by the crypt isolation technique. The mice were sacrificed by cervical dislocation. When the animal showed weight loss (more than 20% in 3 days or more than 25% in a week), they were euthanized.

For DNA methylation analysis, genomic DNA was extracted from the tissues collected as above using a QIAamp DNA Mini Kit (Qiagen), and quantified using a Quant-iT PicoGreen dsDNA Assay Kit (Thermo Fisher Scientific, Waltham, MA). For histological analysis, entire gastric and colonic tissues were fixed by formalin, and embedded in paraffin.

### Genome-wide DNA methylation analysis

Genome-wide DNA methylation profiles were obtained using an Infinium Mouse Methylation BeadChip (Illumina, San Diego, CA), which covered 284,860 CpG sites. Briefly, genomic DNA (500 ng) was treated with sodium bisulfite using an EZ DNA Methylation Kit (Zymo Research, Irvine, CA), and bisulfite-modified DNA was hybridized to the BeadChip array, as described [30]. The BeadChip array was scanned using an iScan system (Illumina), and the obtained data were analyzed using GenomeStudio Methylation Module Software (Illumina). A methylation level of an individual CpG site was represented by a β value ranging from 0 (unmethylated) to 1 (fully methylated). Information of TSS200 and CGIs was obtained from GRCm38/mm10 GENCODE VM23, and that of the enhancer regions was utilized from the atlas of dynamic chromatin landscapes in mice [31].

DNA methylation profiles of human monocytes, CD4^+^ T cells, CD8^+^ T cells, B cells, and NK cells analyzed using an Infinium HumanMethylation450 array (GSE35069) were obtained from the GEO database [32].

### Estimation of the leukocyte fraction by immunofluorescence

The formalin-fixed paraffin-embedded (FFPE) samples were sliced at 4 μm thickness. After deparaffinization, sections were heated in Histofine (pH 9) (Nichirei, Tokyo, Japan) at 121°C for 20 min by an autoclave for antigen retrieval. After washing with 1 x PBS (-) three times, tissue sections were incubated with a primary antibody against mouse Cd45 (1:100, ab10558, Abcam, Cambridge, UK) at room temperature overnight. After washing with 1 x PBS (-) three times, tissue sections were incubated with Alexa Fluor 594-conjugated goat anti-rabbit IgG (1:100, ab 150084, Abcam) at room temperature for 60 min. Sections were co-stained with DAPI. The fluorescence was detected using an all-in-one fluorescence microscope (BZ-X710, KEYENCE, Osaka, Japan), and the number of nuclei (DAPI-stained spots) of epithelial cells and leukocytes, nuclei within Cd45-stained regions, was counted using the BZ-X Analyzer software (KEYENCE). Three independent areas in one stained section were analyzed.

## Results

### The presence of DNA methylation profiles specific to mouse leukocyte lineages

**Fig 1:**
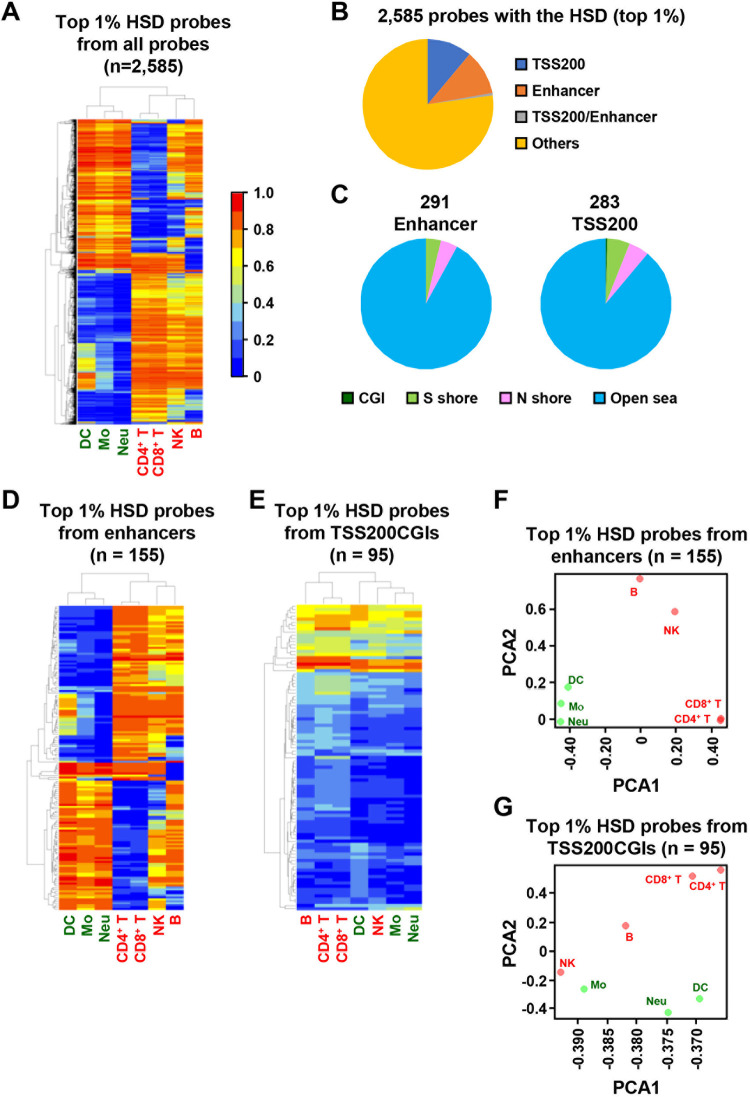
The presence of DNA methylation profiles specific to individual types of mouse leukocytes. (A) Unsupervised hierarchical clustering analysis using the top 1% (2,588 probes) of the total 258,525 probes that had the HSD. The three myeloid lineage cell types and four lymphoid lineage cell types were clustered separately. (B) Origins of the 2,588 probes against gene structure. 291 (11.3%) and 283 (10.9%) of probes were derived from enhancers and TSS200, respectively. (C) Origins of the 291 enhancer and 283 TSS200 probes against CGI. The vast majority of the HSD probes were in open sea, and 0 enhancer probe and 1 TSS200 probe were in CGI. (D) Unsupervised hierarchical clustering analysis using the top 1% (155 probes) from enhancers. The three myeloid lineages and the four lymphoid lineages were classified, similar to the use of the HSD probes from all genomic regions. (E) Unsupervised hierarchical clustering analysis using the top 1% (95 probes) from TSS200CGIs. Separation of the two lineages was not clear. (F) and (G) Principal component analysis (PCA) using the HSD probes from enhancers (F) and those from TSS200 (G). Separation of the two lineages was clearer using probes from enhancers.

Pure cell populations of three myeloid-lineage cells [dendritic cells (16 mice), monocytes (5 mice), and neutrophils (8 mice)] and four lymphoid-lineage cells [CD4^+^ T cells (1 mouse), CD8^+^ T cells (1 mouse), B cells (1 mouse), and natural killer (NK) cells (4 mice)] from mouse spleen were prepared by fluorescence-activated cell sorting (FACS) after magnetic-activated cell sorting (S1 Fig). Since DNA methylation profiles of T cells and B cells among individuals has been reported to be similar [32], cells were isolated from 1 mouse. DNA methylation microarray analysis was conducted using the isolated three myeloid-lineage cell types and four lymphoid-lineage cell types. Clustering analysis was conducted using the top 1% (2,585 probes) with the highest standard deviation (HSD) among the total 258,525 CpG sites (probes). The three myeloid cell types and four lymphoid cell types clustered together, CD4^+^ T cells and CD8^+^ T cells were clustered closely, and B cells and NK cells were clustered together before being clustered with T cells (Fig 1A).

The 2,585 probes contained 283 probes within TSS200 [200 bp-upstream regions from the transcription start sites (TSSs)] and 291 probes within enhancers (Fig 1B), and the vast majority was located outside CGIs (open sea) (Fig 1C). Probes within enhancers were overrepresented considering the total number of the probes within enhancers (15,531 probes) while those within TSS200 were not (total number: 39,012 probes). Clustering analysis using probes within enhancers clearly separated the two lineages (Fig 1D), but did not when using probes within TSS200 located within a CpG island (CGI), the other major regulatory element (Fig 1E). Principal component analysis (PCA) using the probes within enhancers also showed clear separation of myeloid and lymphoid lineages while that using the probes within TSS200CGIs did not (Fig 1F).

**Fig 2:**
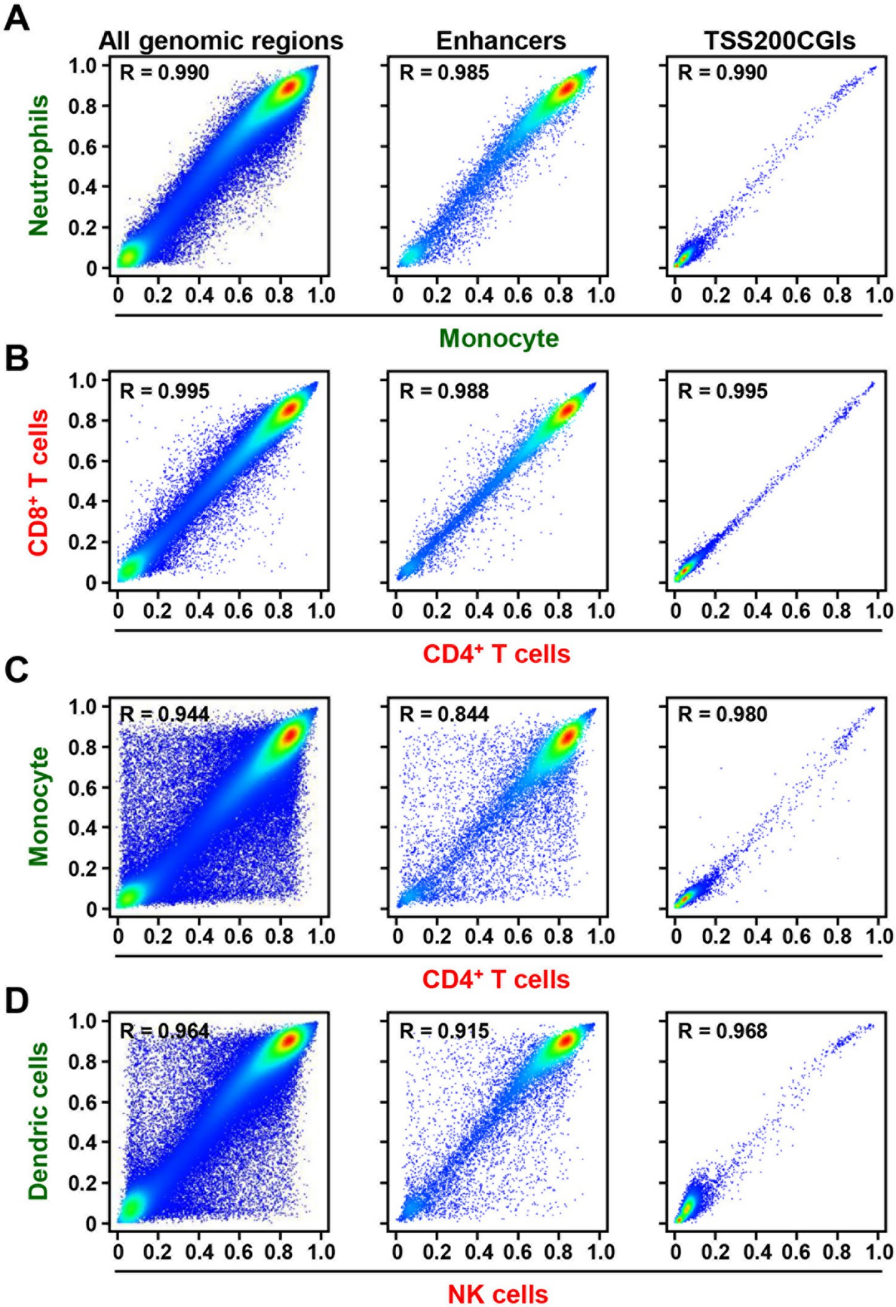
Comparison of methylation levels of all genomic regions, TSS200CGIs, and enhancers between individual types of leukocytes. Using all the genomic regions and the enhancers, methylation profiles were similar between cell types in one cell lineage (A and B), but different between cell types from different cell lineages (C and D). In contrast, methylation profiles were similar even between cell types from different cell lineages in the TSS200CGIs. The x and y axes show DNA methylation levels of individual cell types.

Pair-wise comparison of the DNA methylation levels was made using the total 258,525 probes. DNA methylation profiles were similar (all genomic regions, R = 0.958–0.995) between two cell types within one cell lineage (myeloid or lymphoid) (Fig 2A and 2B), but were highly different (all genomic regions, R = 0.927–0.969) between cell types from different cell lineages (Fig 2C and 2D). The effect of CpG density was further analyzed. Probes in open sea showed high correlations within one cell lineage and differences between different cell lineages, similar to the use of probes in all genomic regions (open sea, R = 0.860–0.941 for different lineages). In contrast, probes in CGIs did not show much difference even between different lineages (CGIs, R = 0.981–0.997) (S2 Fig). These results showed that DNA methylation profiles at CpG sites within enhancers mainly reflected leukocyte cell lineages and types.

### Cell type-specific hypomethylation of genes involved in lineage commitment

**Fig 3:**
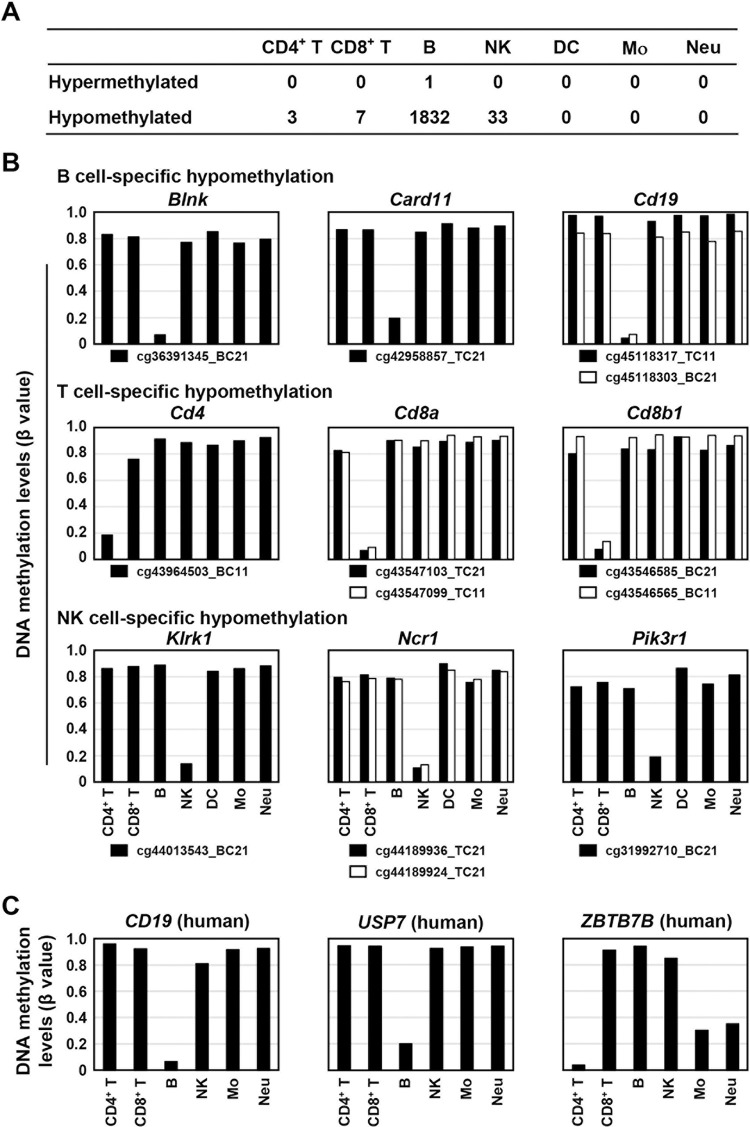
Cell type-specific hypomethylation of genes involved in lineage commitment. (A) Number of CpG sites hypermethylated or hypomethylated in one cell type of leukocytes. One CpG site was specifically hypermethylated in B cells, and three, seven, 182, and 33 CpG sites were specifically hypomethylated in CD4^+^ T cells, CD8^+^ T cells, B cells, and NK cells, respectively. (B) Lineage-specific hypomethylation of CpG sites within genes related to specific types of leukocytes. CpG sites within genes involved in B cell function, *Blnk*, *Card11*, and *Cd19*, were specifically hypomethylated in B cells. CpG sites within genes involved in T cell function, *Cd4a*, Cd8a, and *Cd8b*, were specifically hypomethylated in T cells. CpG sites within genes involved in NK cell function, *Klrk1*, *Ncr1*, and *Pik3r1*, were specifically hypomethylated in NK cells. (C) Human relevance of lineage-specific hypomethylation. The leukocyte cell type-specific hypomethylation of *Cd19*, *Usp7*, and *Zbtb7b* in the mouse genome was conserved at their homologous regions in the human genome.

To isolate lineage-specific methylated or unmethylated CpG sites, CpG sites hypermethylated (β value ≥ 0.7) or hypomethylated (β value < 0.2) only in all the cell types within one lineage were searched for. 771 and 2,254 CpG sites were specifically methylated and unmethylated, respectively, in the myeloid lineage cell types. 1,284 and 211 of the total 258,525 CpG sites were specifically methylated and unmethylated, respectively, in the lymphoid lineage cell types. Then, cell-type specifically methylated or unmethylated CpG sites were searched for. Specific hypermethylation was observed only in one CpG site for B cells, and specific hypomethylation was observed in three, seven, 182, and 33 CpG sites for CD4^+^ T cells, CD8^+^ T cells, B cells, and NK cells, respectively (Fig 3A).

The 182 CpG sites specifically hypomethylated in B cells were located at genes, including those involved in B cell functions, such as *Blnk*, *Card11*, and *Cd19*, while the one hypermethylated CpG site was located at a gene not related to B cell function, *Fmnl2*. The three and seven CpG sites specifically hypomethylated in CD4^+^ and CD8^+^ T cells, respectively, were located at genes, including those involved in T cell functions, such as *Cd4*, *Cd8a*, and *Cd8b1*. The 33 CpG sites hypomethylated specifically in NK cells were located at genes, including those involved in NK cell functions, such as *Klrk1*, *Ncr1*, and *Pik3r1* (Fig 3B).

To analyze human relevance, DNA methylation statuses of human CpG sites corresponding to the CpG sites specifically methylated or unmethylated in mice were analyzed using a database for human leukocytes. It was revealed that 159 of 226 CpG sites were located at homologous regions in humans, and, for 15 of 159 CpG sites, DNA methylation data obtained by human DNA methylation microarray were available [32]. Among the 15 CpG sites, three CpG sites were also hypomethylated in specific types of human leukocytes (Fig 3C). This result indicated that cell type-specific hypomethylation and hypermethylation was important across species, and that they may be located at genes involved in the development and functions of specific leukocytes.

### Establishment of an algorithm to estimate the leukocyte fraction, and its successful use in inflamed gastrointestinal tissues

We proceeded to establish an algorithm with the DNA methylation levels of specific CpG sites to estimate the fraction of specific cell types in a heterogeneous tissue sample, such as the one exposed to chronic inflammation. Since the leukocytes infiltrate epithelial layers by chronic inflammation, we utilized gastric epithelia isolated by scraping and colonic epithelia isolated by crypt isolation. To this end, we first isolated marker CpG sites highly methylated (β value ≥ 0.9) in two leukocyte samples, but not (top 50 CpG sites with lowest methylation levels) in gastric (n = 4) or colonic (n = 2) epithelium samples (S3A and S3B Fig). To estimate a fraction of leukocytes in a DNA sample, observed methylation difference of the 50 marker CpG sites between tissues with and without leukocyte infiltration [Δβ value (inflamed epithelium–non-inflamed epithelium); y] was plotted against ideal difference [Δβ value (leukocytes–non-inflamed epithelium); x]. Since CpG sites which showed higher methylation levels were potentially methylated also in epithelium and affected by chronic inflammation (S3C Fig), 40 CpG sites with the lowest Δβ values of the 50 marker CpG sites (y-axis), were used for drawing a regression line. The slope of the regression line was considered as the leukocyte fraction of an individual sample, as established in humans [27].

**Fig 4:**
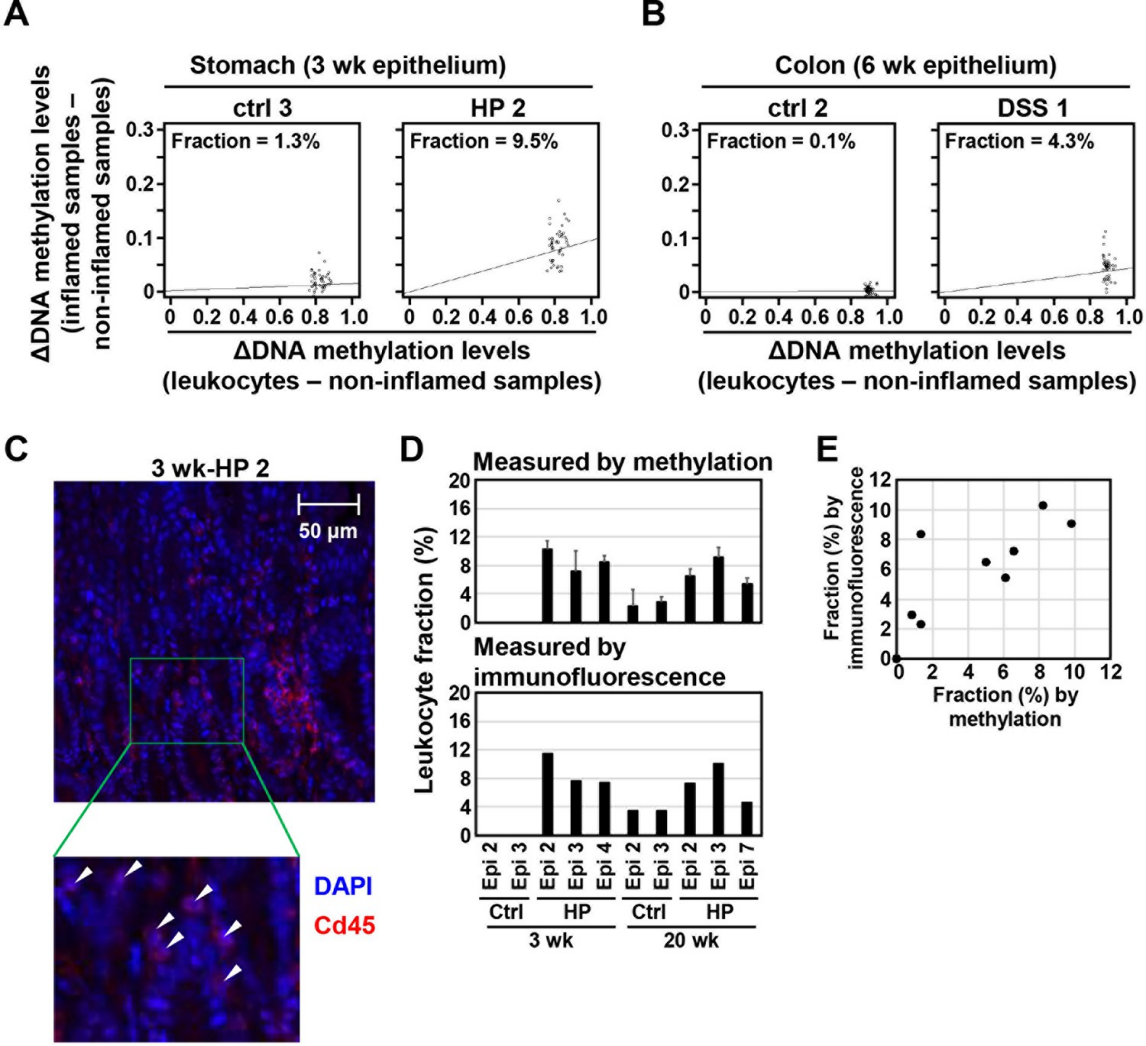
Estimation of leukocyte fraction in inflamed tissues by an algorithm using leukocyte-specific DNA methylation. (A) and (B) Estimation of leukocyte fraction in *H*. *pylori*-infected stomach (A) and DSS-treated colon (B). The fraction of infiltrating leukocytes was 7.8% in *H*. *pylori*-infected stomach (HP 2) and 4.3% in DSS-treated colon (DSS 1), but 1.3% and 0.1% in their corresponding control samples (ctrl 3 and ctrl 2). (C) Histological estimation of a leukocyte fraction. The fraction of infiltrating leukocytes was histologically estimated by immunofluorescence of a leukocyte marker, Cd45, and DAPI staining. The number of DAPI-stained spots within Cd45-stained regions was counted as that of nuclei of leukocytes. White arrowheads show leukocytes. (D) and (E) The fraction of infiltrating leukocytes in *H*. *pylori*-infected stomach estimated using the algorithm with DNA methylation and that obtained histologically. The fraction in infected stomach was 7.6±3.6% and 7.8±1.6% using methylation markers and immunofluorescence, respectively (D). The fraction estimated using two methods was well correlated [R = 0.811 (*p* = 0.004)] (E).

Using this algorithm involving the 50 marker CpG sites informative in the stomach and colon (S1 and S2 Tables), leukocyte fractions in the inflamed stomach and colon were estimated. The algorithm estimated the fraction of infiltrating leukocytes at 1.6–12.4% in six samples of inflamed stomach (Fig 4A, S4A and S4B Fig), and at 1.5 and 4.3% in two samples of inflamed colon (Fig 4B, and S4C Fig). The fraction estimated by methylation markers was highly correlated with that histologically estimated using Cd45-stained tissue sections [R = 0.811 (*p* = 0.004)] in 10 stomach samples (Fig 4C–4E, and S5 Fig). This result showed that the marker CpG sites can be utilized for the estimation of the fraction of leukocytes infiltrating a tissue.

## Discussion

Lineage-specific DNA methylation was successfully isolated for mouse myeloid and lymphoid cells. In addition to the HSD probes among the total probes, those in enhancer regions successfully separated the three myeloid cell types and the four lymphoid cell types. The CD4^+^ T and CD8^+^ T cells were clustered closely, and the B and NK cells were clustered before being clustered with T cells. The phylogenic tree was highly consistent with the tree established for mouse and human leukocytes [33]. In contrast, probes within CGIs failed to separate lineages, and this was in line with the fact that tissue- or cell-type specific DMRs are located at gene regulatory elements, such as enhancers [7, 23], and enhancers frequently located outside of CGIs.

Cell type-specific hypomethylated CpG sites were located at genes related to their functions. *Blnk* (*SLP65*), which contained a B cell-specifically hypomethylated CpG site, is known to be involved in the recombination of a gene encoding immunoglobulin light chain and pre-B cell differentiation [34, 35]. *Klrk1* (*NKG2D*), which contained a NK cell-specifically hypomethylated CpG site, is known to be involved in the cytokine secretion from NK cells [36–38]. However, different from gene silencing due to DNA methylation of promoter CGIs, the roles of the hypomethylation of the CpG sites in transcriptional regulation of respective genes need to be carefully established. Importantly, some of the leukocyte cell type-specific hypomethylation in the mouse genome was conserved at the homologous regions in the human genome.

An algorithm to estimate the fraction of mouse leukocytes infiltrating the inflamed tissues was established using 50 CpG sites highly methylated in leukocytes and unmethylated in the tissues, and high correlation [R = 0.811 (*p* = 0.004)] between the fraction assessed by the algorithm and that by histology was shown. To use 40 CpG sites with the lowest Δβ values among the 50 CpG sites enabled us to remove probes whose methylation levels were highly affected by chronic inflammation in individual samples. The estimation of leukocyte fraction by the algorithm can be applied to samples of which only DNA samples are available, which is a strong advantage over FACS and single-cell analyses. Also in human tissues, the usefulness of this strategy has been reported in our previous study [27]. The strategy will become more cost-effective if the number of CpG sites used for the estimation can be further reduced and locus-specific DNA methylation analysis is developed.

The pure populations of leukocytes were isolated from splenocytes in this study. This raises a concern that their methylation profiles could be different from those in gastrointestinal tissues. However, the high correlation between the fraction assessed by the algorithm and that by histological analysis cleared this concern, at least for the CpG sites used for the algorithm to estimate a leukocyte fraction.

## Conclusions

DNA methylation profiles at CpG sites located in enhancers reflected mouse leukocyte cell lineage, and the use of the marker CpG sites can estimate leukocyte fractions within inflamed tissues.

## Supporting information

S1 Fig Fraction of individual cell types among whole leukocytes. (A) The fraction among myeloid-lineage cells. Dendric cells, monocytes, and neutrophils were separated by MACS. (B) The fraction among lymphoid-lineage cells. CD4^+^/CD8^+^ T cell, B cells, and NK cells were separated by MACS.
(PDF)

S2 Fig Comparison of two cell types using methylation levels of CpG sites within open sea and CGIs. Using CpG sites within open sea, methylation profiles were similar between cell types from one cell lineage (A and B), but different between cell types from different cell lineages (C and D). In contrast, using CpG sites within the CGIs, methylation profiles were similar even between cell types from different cell lineages.
(PDF)

S3 Fig Selection of CpG sites methylated in all types of leukocytes and unmethylated in gastrointestinal tissues (probes). (A) From the total 258,525 CpG sites, 14,186 CpG sites highly methylated (β ≥ 0.9) in the total leukocyte samples (n = 2) were selected. From these 14,186 CpG sites, the top 50 CpG sites with lowest methylation levels in the gastric epithelium (average methylation levels of 4 mice) or colonic epithelium (average methylation levels of 2 mice) were selected as leukocyte fraction markers in the stomach and colon, respectively. (B) DNA methylation levels of isolated marker CpG sites in individual types of leukocytes. Marker probes were also highly methylated in all the individual cell types of leukocytes. (C) To estimate a fraction of leukocytes in a DNA sample, DNA methylation levels of 50 CpG markers were plotted [x = Δβ value (leukocytes–non-inflamed epithelium); y = Δβ value (inflamed epithelium–non-inflamed epithelium)]. The 50 CpG markers can be classified into 1) markers whose methylation levels were not affected by chronic inflammation (left) and 2) markers whose methylation levels were affected by chronic inflammation (middle). Therefore, a regression line whose slope was considered as the leukocyte fraction was drawn using markers not affected by chronic inflammation (right). 40 of 50 CpG markers were utilized as those not affected by chronic inflammation.
(PDF)

S4 Fig Estimation of a leukocyte fraction in the stomach infected by *H*. *pylori* for three weeks. (A) and 20 weeks (B) and in the DSS-treated colon (C). The fraction of infiltrating leukocytes was 1.4–9.8% in the *H*. *pylori*-infected stomach, but 0–1.4% in corresponding control samples.
(PDF)

S5 Fig Diagrammatic summary of the establishment and validation of an estimation algorithm using methylation marker CpG sites to estimate the leukocyte fraction. (PDF)

S1 Table 50 CpG sites used for the estimation of the fraction of infiltrating leukocytes in the stomach. (PDF)

S2 Table 50 CpG sites used for the estimation of the fraction of infiltrating leukocytes in the colon. (PDF)

## List of abbreviations

*H*. *pylori*: *Helicobacter pylori*
DSS: dextran sulfate sodium
DMR: differentially methylated region
NK cells: natural killer cells
CGI: CpG island
