# Supplementary material for: Mouse methylation profiles for leukocyte cell types, and estimation of leukocyte fractions in inflamed gastrointestinal DNA samples
Source: PLoS One. 2023 Oct 5;18(10):e0290034. doi: 10.1371/journal.pone.0290034 (PMC10553802; doi:10.1371/journal.pone.0290034)
Supplement: S1 Fig — (A) The fraction among myeloid-lineage cells. Dendric cells, monocytes, and neutrophils were separated by MACS. (B) The fraction among lymphoid-lineage cells. CD4+/CD8+ T cell, B cells, and NK cells were separated by MACS. (PDF) [file pone.0290034.s001.pdf]

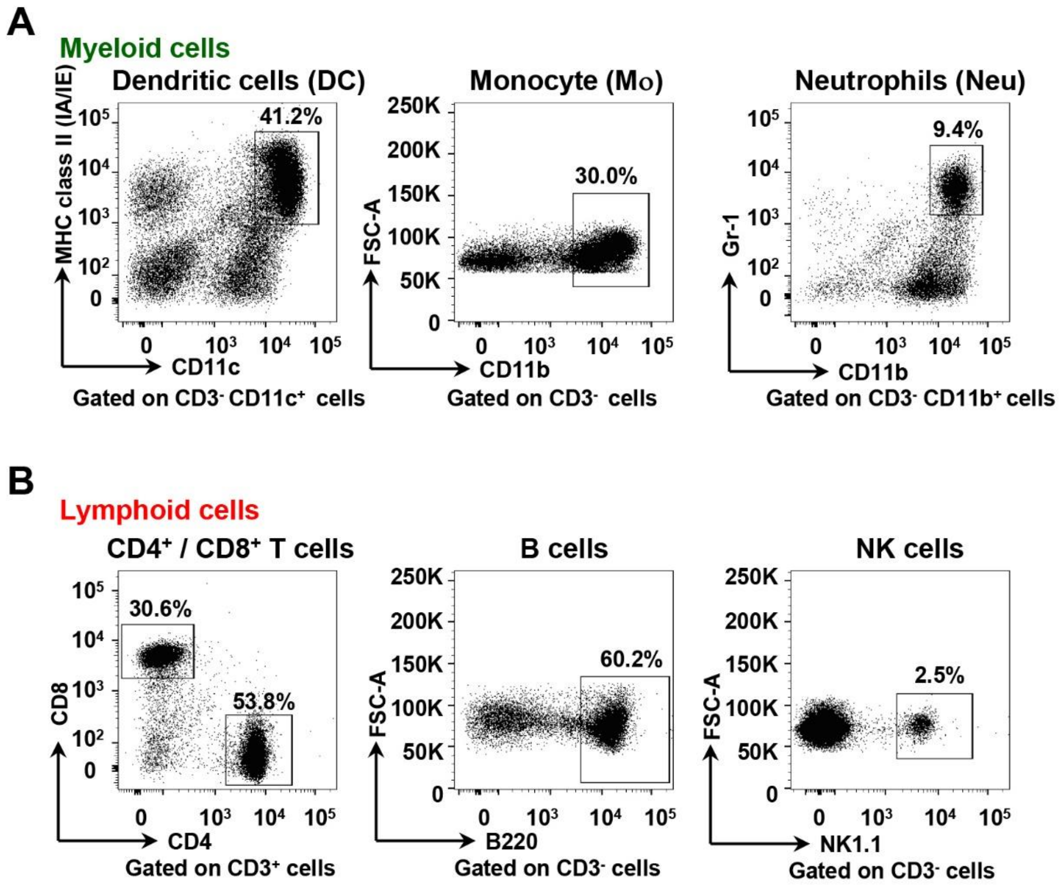

S1 Fig.

Fraction of individual cell types among whole leukocytes. (A) The fraction among myeloid-lineage cells. Dendric cells, monocytes, and neutrophils were separated by MACS. (B) The fraction among lymphoid-lineage cells. $CD4^+/CD8^+$ T cell, B cells, and NK cells were separated by MACS.
