# Supplementary material for: Mouse methylation profiles for leukocyte cell types, and estimation of leukocyte fractions in inflamed gastrointestinal DNA samples
Source: PLoS One. 2023 Oct 5;18(10):e0290034. doi: 10.1371/journal.pone.0290034 (PMC10553802; doi:10.1371/journal.pone.0290034)
Supplement: S2 Fig — Using CpG sites within open sea, methylation profiles were similar between cell types from one cell lineage (A and B), but different between cell types from different cell lineages (C and D). In contrast, using CpG sites within the CGIs, methylation profiles were similar even between cell types from different cell lineages. (PDF) [file pone.0290034.s002.pdf]

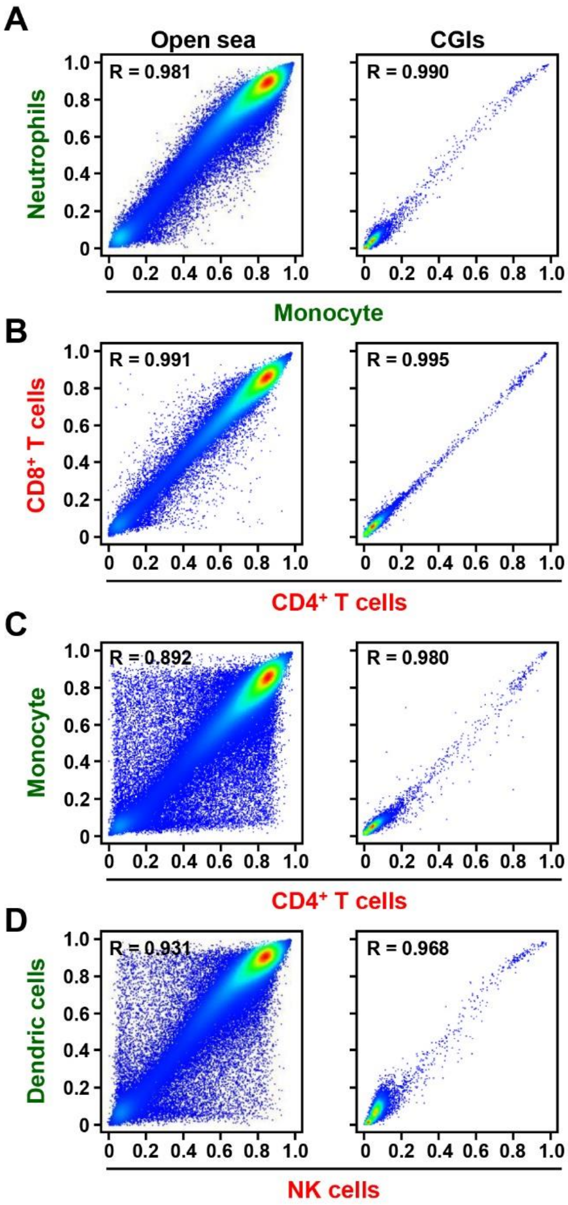

S2 Fig.

Comparison of two cell types using methylation levels of CpG sites within open sea and CGIs. Using CpG sites within open sea, methylation profiles were similar between cell types from one cell lineage (A and B), but different between cell types from different cell lineages (C and D). In contrast, using CpG sites within the CGIs, methylation profiles were similar even between cell types from different cell lineages.
