# Supplementary material for: Mouse methylation profiles for leukocyte cell types, and estimation of leukocyte fractions in inflamed gastrointestinal DNA samples
Source: PLoS One. 2023 Oct 5;18(10):e0290034. doi: 10.1371/journal.pone.0290034 (PMC10553802; doi:10.1371/journal.pone.0290034)
Supplement: S3 Fig — (A) From the total 258,525 CpG sites, 14,186 CpG sites highly methylated (β ≥ 0.9) in the total leukocyte samples (n = 2) were selected. From these 14,186 CpG sites, the top 50 CpG sites with lowest methylation levels in the gastric epithelium (average methylation levels of 4 mice) or colonic epithelium (average methylation levels of 2 mice) were selected as leukocyte fraction markers in the stomach and colon, respectively. (B) DNA methylation levels of isolated marker CpG sites in individual types of leukocytes. Marker probes were also highly methylated in all the individual cell types of leukocytes. (C) To estimate a fraction of leukocytes in a DNA sample, DNA methylation levels of 50 CpG markers were plotted [x = Δβ value (leukocytes–non-inflamed epithelium); y = Δβ value (inflamed epithelium–non-inflamed epithelium)]. The 50 CpG markers can be classified into 1) markers whose methylation levels were not affected by chronic inflammation (left) and 2) markers whose methylation levels were affected by chronic inflammation (middle). Therefore, a regression line whose slope was considered as the leukocyte fraction was drawn using markers not affected by chronic inflammation (right). 40 of 50 CpG markers were utilized as those not affected by chronic inflammation. (PDF) [file pone.0290034.s003.pdf]

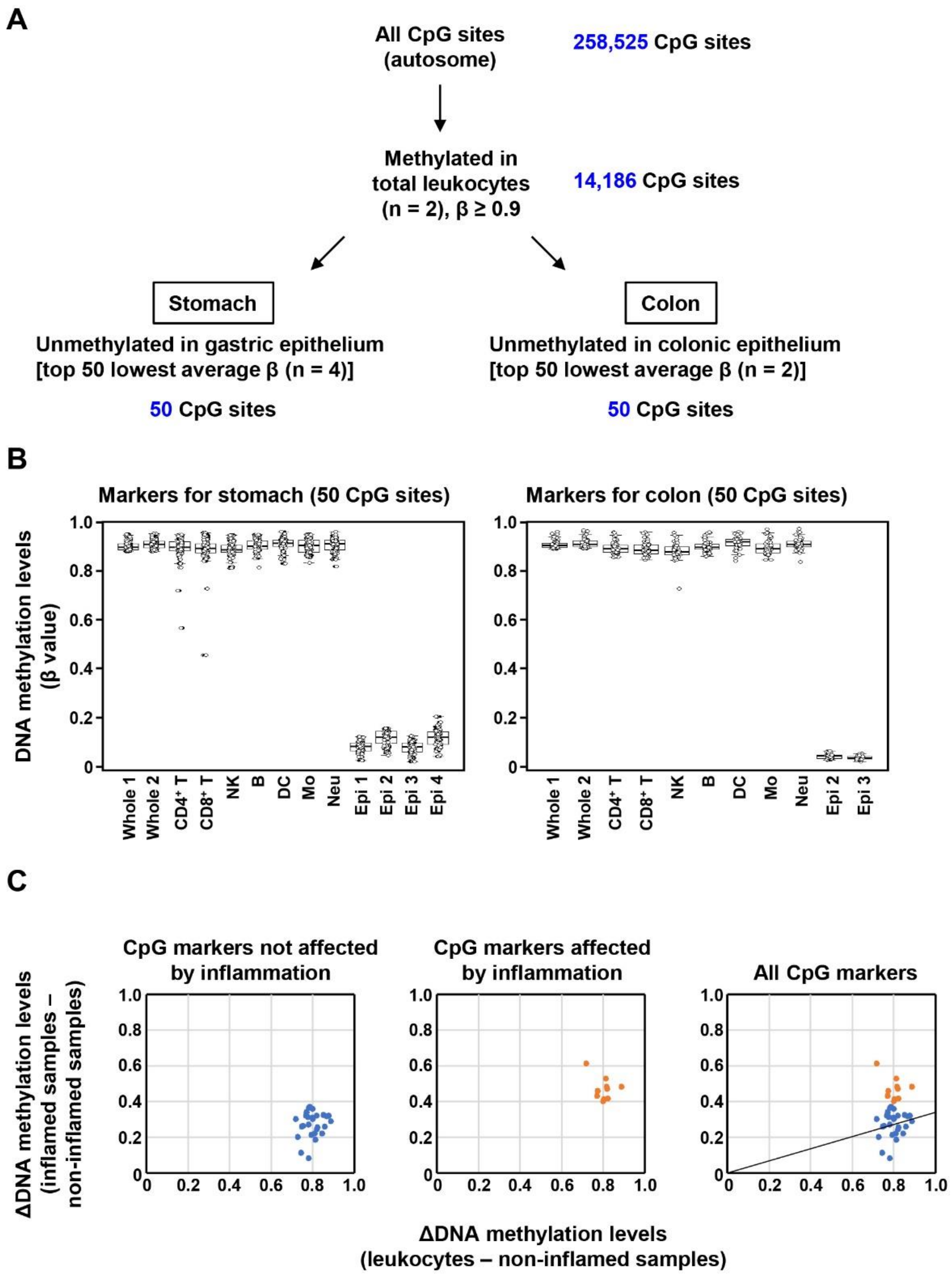

S3 Fig.

Selection of CpG sites methylated in all types of leukocytes and unmethylated in gastrointestinal tissues (probes). (A) From the total 258,525 CpG sites, 14,186 CpG sites

highly methylated ($\beta \geq 0.9$) in the total leukocyte samples (n = 2) were selected. From these 14,186 CpG sites, the top 50 CpG sites with lowest methylation levels in the gastric epithelium (average methylation levels of 4 mice) or colonic epithelium (average methylation levels of 2 mice) were selected as leukocyte fraction markers in the stomach and colon, respectively. (B) DNA methylation levels of isolated marker CpG sites in individual types of leukocytes. Marker probes were also highly methylated in all the individual cell types of leukocytes. (C) To estimate a fraction of leukocytes in a DNA sample, DNA methylation levels of 50 CpG markers were plotted [x = $\Delta\beta$ value (leukocytes – non-inflamed epithelium); y = $\Delta\beta$ value (inflamed epithelium – non-inflamed epithelium)]. The 50 CpG markers can be classified into 1) markers whose methylation levels were not affected by chronic inflammation (left) and 2) markers whose methylation levels were affected by chronic inflammation (middle). Therefore, a regression line whose slope was considered as the leukocyte fraction was drawn using markers not affected by chronic inflammation (right). 40 of 50 CpG markers were utilized as those not affected by chronic inflammation.
