# Supplementary material for: Mouse methylation profiles for leukocyte cell types, and estimation of leukocyte fractions in inflamed gastrointestinal DNA samples
Source: PLoS One. 2023 Oct 5;18(10):e0290034. doi: 10.1371/journal.pone.0290034 (PMC10553802; doi:10.1371/journal.pone.0290034)
Supplement: S4 Fig — (A) and 20 weeks (B) and in the DSS-treated colon (C). The fraction of infiltrating leukocytes was 1.4–9.8% in the H. pylori-infected stomach, but 0–1.4% in corresponding control samples. (PDF) [file pone.0290034.s004.pdf]

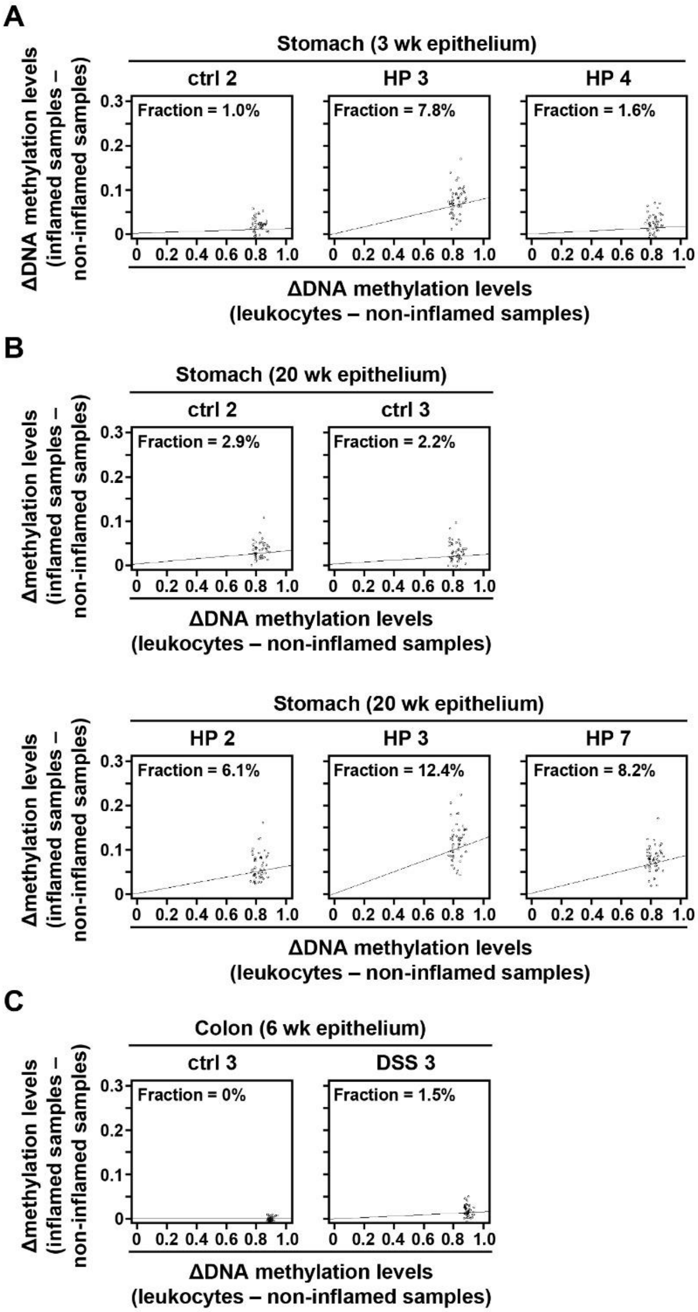

S4 Fig.

Estimation of a leukocyte fraction in the stomach infected by *H. pylori* for three weeks (A) and 20 weeks (B) and in the DSS-treated colon (C). The fraction of infiltrating leukocytes was 1.4-9.8% in the *H. pylori*-infected stomach, but 0-1.4% in corresponding control samples.
