# Supplementary material for: Mouse methylation profiles for leukocyte cell types, and estimation of leukocyte fractions in inflamed gastrointestinal DNA samples
Source: PLoS One. 2023 Oct 5;18(10):e0290034. doi: 10.1371/journal.pone.0290034 (PMC10553802; doi:10.1371/journal.pone.0290034)
Supplement: S5 Fig — (PDF) [file pone.0290034.s005.pdf]

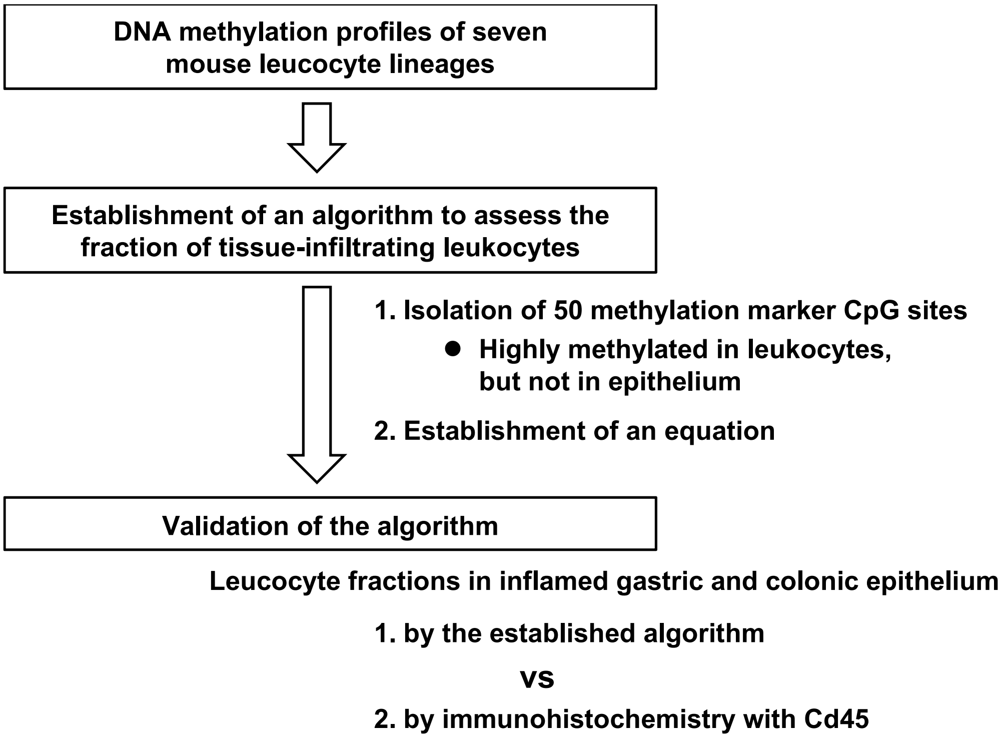


## S5 Fig.

Diagrammatic summary of the establishment and validation of an estimation algorithm using methylation marker CpG sites to estimate the leukocyte fraction.
