# Supplementary material for: Mouse methylation profiles for leukocyte cell types, and estimation of leukocyte fractions in inflamed gastrointestinal DNA samples
Source: PLoS One. 2023 Oct 5;18(10):e0290034. doi: 10.1371/journal.pone.0290034 (PMC10553802; doi:10.1371/journal.pone.0290034)
Supplement: S1 Table — (PDF) [file pone.0290034.s006.pdf]

S1 Table. 50 CpG sites used for the estimation of the fraction of infiltrating leukocytes in the stomach.

Target ID	Chr	Position
cg36848905_TC11	1	40140081
cg37203122_BC11	1	89072500
cg38282601_BC21	2	35692102
cg38425539_TC11	2	59179234
cg38842623_BC11	2	117086138
cg39122572_BC11	2	152785631
cg40463486_TC11	3	146770057
cg41165791_TC11	4	97780124
cg41999154_TC21	5	32141206
cg42136919_TC11	5	51591711
cg43270580_BC11	6	31321994
cg43394535_BC21	6	49097235
cg43763259_TC21	6	99101625
cg44037310_TC21	6	134263409
cg44129566_BC11	6	144056975
cg44516613_BC11	7	45234605
cg44541537_TC11	7	46861230
cg44920641_TC21	7	100943203
cg45136308_BC11	7	127691822
cg46232196_TC21	8	117339568
cg46364185_TC11	8	126525025
cg46843185_TO21	9	61997996
cg46854722_TC11	9	63091510
cg46918552_BC11	9	69457853
cg46966062_BC11	9	74909477
cg47346773_BC11	9	118939631
cg47346774_TC11	9	118939647
cg28150184_BC11	10	8213398
cg28150186_TC11	10	8213582
cg28207572_BC11	10	15760141
cg28381836_TC11	10	40857783
cg28579406_BC11	10	66922144
cg28593935_TC11	10	68127990
cg28879104_BC11	10	95717495

S1 Table. continued.

Target ID	Chr	Position
cg29089535_BC11	10	121510420
cg29551928_TC11	11	54004470
cg29720960_BC21	11	70466090
cg29903391_BC11	11	88394725
cg29979572_BC11	11	96345972
cg30121845_BC11	11	107337627
cg31999181_TC11	13	102590227
cg32162476_BC11	14	7952786
cg33759456_TC11	15	97376994
cg34843747_BC11	17	32392621
cg35259852_TC21	17	80802671
cg35321883_TC11	17	86780832
cg35449337_TC11	18	13752113
cg35880862_TC11	18	69412892
cg36172850_BC11	19	14424519
cg36521594_TC11	19	53517470
