# Supplementary material for: Mouse methylation profiles for leukocyte cell types, and estimation of leukocyte fractions in inflamed gastrointestinal DNA samples
Source: PLoS One. 2023 Oct 5;18(10):e0290034. doi: 10.1371/journal.pone.0290034 (PMC10553802; doi:10.1371/journal.pone.0290034)
Supplement: S2 Table — (PDF) [file pone.0290034.s007.pdf]

S2 Table. 50 CpG sites used for the estimation of the fraction of infiltrating leukocytes in the colon.

Target ID	Chr	Position
cg36799961_TC11	1	35931161
cg37062550_BC21	1	71837879
cg37254154_TC21	1	93731890
cg37710891_TC11	1	164455315
cg37971074_TC21	1	191741894
cg38282601_BC21	2	35692102
cg39122572_BC11	2	152785631
cg39403390_TC11	2	178643327
cg39994608_BC21	3	88496298
cg40940692_TC11	4	58563453
cg41315512_BC21	4	116406035
cg42393084_TC21	5	89219091
cg42466068_BC21	5	99329099
cg42515217_BC21	5	103821328
cg43000082_BC21	5	144342107
cg43261574_TC21	6	30477630
cg43394535_BC21	6	49097235
cg43649921_BC21	6	86233338
cg43763259_TC21	6	99101625
cg44734127_BC21	7	75415716
cg45141397_TC11	7	128032989
cg45894898_TC21	8	80853792
cg46744723_BC21	9	51312668
cg46843185_TO21	9	61997996
cg46854722_TC11	9	63091510
cg46918552_BC11	9	69457853
cg46966062_BC11	9	74909477
cg28185305_BC21	10	12853278
cg28225140_TC21	10	18463561
cg28579406_BC11	10	66922144
cg28879104_BC11	10	95717495
cg28912610_BC11	10	100042685
cg29551928_TC11	11	54004470
cg29921382_TC21	11	89997113

S2 Table. continued.

Target ID	Chr	Position
cg30216208_BC11	11	115738784
cg30282083_BC21	11	119945685
cg31686928_TC11	13	59454229
cg31716718_BC21	13	63156326
cg31784497_TC11	13	72198729
cg32013322_TC21	13	104037189
cg33040530_TC21	15	7015259
cg33066706_TC21	15	10215896
cg33820293_BC21	15	101370167
cg34596776_TC21	17	6728294
cg34653758_TC21	17	12792548
cg34680107_BC21	17	15824570
cg34780684_BC21	17	27810676
cg36381747_TC21	19	40187403
cg36425647_BC21	19	43889186
cg36521594_TC11	19	53517470
